# Formative Development and Acceptability of a Lifestyle Weight Management Intervention for Breast Cancer Survivors in Greece: The NutriLife Study

**DOI:** 10.3390/healthcare13141683

**Published:** 2025-07-12

**Authors:** Maria Perperidi, Eleni Skeparnakou, Dimitra Strongylou, Ariadni Leptopoulou, Thomas Tsiampalis, Konstantinos Tsapakidis, Emmanouil Saloustros, Yannis Theodorakis, Odysseas Androutsos

**Affiliations:** 1 Laboratory of Clinical Nutrition and Dietetics, Department of Nutrition and Dietetics, School of Physical Education, Sport Science and Dietetics, University of Thessaly, 42132 Trikala, Greece; mperperidi@uth.gr (M.P.); dstrongylou@uth.gr (D.S.); ariadni.leptopoulou@gmail.com (A.L.); ttsiampalis@uth.gr (T.T.); 2 Oncology Unit, 3rd Department of Medicine, ‘Sotiria’ General Hospital, National and Kapodistrian University of Athens, 11527 Athens, Greece; eleniskeparnakou@gmail.com; 3 Department of Oncology, Medical School, University Hospital of Larissa, 41334 Larissa, Greece; tsapakidisk@yahoo.com (K.T.); esaloustros@uth.gr (E.S.); 4 Department of Physical Education and Sport Science, School of Physical Education, Sport Science and Dietetics, University of Thessaly, 42132 Trikala, Greece; theodorakis@uth.gr

**Keywords:** breast cancer survivors, behavioral intervention, digital tools, weight management, study design, theoretical framework of acceptability

## Abstract

**Background/Objectives:** Weight gain is frequently observed during and following breast cancer therapy. Women with overweight/obesity have poorer breast cancer prognoses and are more likely to develop comorbidities. The present study describes the development and qualitative assessment of the acceptability of the NutriLife study, a lifestyle weight management intervention with dietetic counseling and digital tools for breast cancer survivors (BCSs). **Methods:** The intervention was developed using the Medical Research Council (MRC) framework, informed by a systematic literature review and stakeholder input. Acceptability was assessed using the Theoretical Framework of Acceptability (TFA). A total of 22 BCSs with overweight/obesity participated in focus groups, and 5 dietitians/nutritionists specializing in breast cancer in Greece participated in semi-structured interviews. The data were further analyzed using thematic analysis. **Results:** Stakeholders assessed the intervention as acceptable across all TFA constructs. The intervention was characterized as supportive, easily adaptable, time-efficient, well-organized, beneficial, and professionally driven, with potential barriers including limited personal time, inadequate digital literacy, insufficient self-care, and lack of commitment. Gradually increasing goals may be helpful and less stressful, while educational resources enhance focus on these objectives, thus encouraging intervention participation. Ensuring confidentiality was perceived as central to promoting health. **Conclusions:** The evidence-based, co-participatory design of the NutriLife intervention was perceived as acceptable by the participating stakeholders and will be pilot-tested in a randomized controlled trial.

## 1. Introduction

A growing body of evidence confirms that obesity is a recognized risk factor for the initiation, progression, and metastasis of breast cancer [1,2,3]. Obesity appears to increase the risk of breast cancer and its recurrence by raising oestrogen levels, promoting inflammation, and contributing to the development of metabolic syndrome [4]. A meta-analysis of 82 studies including over 200,000 patients revealed that the mortality risk increased by 75% in premenopausal women and by 34% in postmenopausal women if they were living with obesity at the time of breast cancer diagnosis, compared to patients with normal body weight [5]. Breast cancer survivors often gain weight, which is linked to a higher risk of comorbid conditions such as type 2 diabetes and cardiovascular disease [6]. A meta-analysis encompassing 12 studies with a total of 23,832 women showed that women who increased their initial body weight by at least 5% after their post-breast cancer diagnosis were at increased risk of mortality from all causes compared to women whose body weight remained stable (HR, 1.12; 95% CI, 1.03–1.22). In fact, mortality risk was highest among patients who experienced a body weight increase of at least 10% [7].

Although the association between intentional weight loss and survival outcomes following primary breast cancer treatment remains unclear, weight loss may reduce the risk of comorbidities. Several leading health authorities recommend managing excess weight in individuals living with or surviving cancer [1,8]. Strategies for managing body weight include preventing weight gain, pursuing intentional weight loss, and maintaining weight loss, emphasizing the importance of counseling to provide ongoing support for behavioral changes among survivors [9,10]. Recent studies indicate that effective weight gain prevention or weight loss interventions must integrate structured physical activity and dietary modifications and should be based on evidence-based behavior change theories and techniques [11,12,13]. These interventions should tackle perceived barriers to weight loss, such as symptoms from breast cancer treatment and fatigue, thereby addressing the unique needs of this population [14].

In order to minimize the impact of obesity on breast cancer, interventions must be complex yet pragmatic in design [11], ensuring that they are acceptable and equally accessible to all women [15]. An intervention may also incorporate existing evidence and/or experience. The study design of such interventions needs to be tailored according to the research questions and the selected outcome measures [16]. It is recommended to integrate appropriate theories or theoretical models and frameworks at the study design stage, drawing on existing evidence and theoretical frameworks from relevant disciplines while engaging different stakeholders. This concept should be refined throughout the research process to yield an updated program theory upon project completion [16]. The Medical Research Council (MRC) and the National Institute for Health Research proposed a new framework that considers recent developments in theory and methods, aiming to enhance the efficiency, utilization, and impact of research [16]. This framework seeks evidence that the key components are addressed in the research design and implementation.

‘Intervention acceptability’, that is, the acceptance of the intervention by both intervention deliverers (e.g., dietitians/nutritionists) and recipients (e.g., breast cancer survivors) constitutes an essential prerequisite for successful intervention implementation [17]. Therefore, intervention acceptability should be considered in the design of healthcare interventions, particularly in contexts characterized by complexity and multiple interacting components [18].

The present study represents the formative phase of the NutriLife intervention and aligns with the updated Medical Research Council (MRC) framework for developing and evaluating complex interventions. While prior studies have addressed weight management in breast cancer survivors, few have integrated theory-based frameworks (MRC, TFA), stakeholder co-creation, and hybrid delivery formats (in-person, group, and digital) within a culturally specific Mediterranean context. This manuscript focuses on the development process and, critically, the qualitative evaluation of the intervention’s acceptability among key stakeholders—namely, breast cancer survivors and oncology-specialized dietitians/nutritionists. A randomized controlled trial has been registered (NCT06577545) and will assess the feasibility, adherence, and preliminary effectiveness of the intervention in the next research phase. The primary aim of the current study is to assess the perceived acceptability of the NutriLife intervention using qualitative methods, in accordance with the Theoretical Framework of Acceptability (TFA). By capturing end-user perspectives, this study aims to inform future adaptations and support the intervention’s implementation readiness.

## 2. Methods

### 2.1. Development of the NutriLife Intervention

The NutriLife intervention is a lifestyle weight management intervention with dietetic counseling and digital tools for breast cancer survivors living with overweight/obesity. It was guided by the Medical Research Council (MRC) framework for the development and evaluation of complex interventions [16], designed by a multidisciplinary group of researchers (dietitians/nutritionists, oncologists, physical education instructors, and clinical and health psychologists) and co-created by stakeholders (breast cancer survivors and dietitians/nutritionists). The MRC framework divides complex intervention research into four phases: development or identification of the intervention, feasibility, evaluation, and implementation. Accordingly, the design of the NutriLife intervention was developed based on a systematic review and by using a co-participatory approach with breast cancer survivors and dieticians/nutritionists specializing in cancer care. The systematic review was conducted to identify the effective behavioral change techniques in nutrition and physical activity interventions for treating overweight or obesity in post-treatment breast cancer survivors [19]. The behavioral change techniques found to be the most effective were the main constructs of the intervention’s context. Furthermore, a qualitative study (focus groups) was conducted to examine the barriers and facilitators influencing breast cancer survivors’ adoption of a healthy lifestyle, as well as to gather their recommendations for the development of weight management interventions [20]. Interviews with dietitians/nutritionists specializing in cancer care were held to evaluate their views and perspectives influencing lifestyle and weight management in breast cancer survivors [21]. The findings of these three preparatory studies were combined to develop an evidence-based intervention for treating overweight/obesity in breast cancer survivors. The co-participatory approach used in its design is expected to increase its potential effectiveness. Prior to the implementation of the NutriLife intervention, the feasibility of the intervention’s design and the evaluation of the intervention’s content were assessed through an acceptability study, as described below.

#### 2.1.1. The NutriLife Intervention: Duration, Content, Target Population, and Deliverers

Breast cancer survivors are defined as women who have received a diagnosis of breast cancer and have completed overall treatment, including surgery, chemotherapy, radiotherapy, and other cancer treatments, excluding hormone or immune therapy.

The study participants in both arms will consist of female breast cancer survivors at least 3 months post-treatment (including surgery, chemotherapy, radiotherapy, or other cancer treatments) and without active cancer therapy or ongoing treatment, except for hormonal or immune therapy. The participants will be aged 18–65 years, have a BMI of 25–40 kg/m^2^, and will not adhere to a specific diet plan during the study period. Everybody must have access to a computer or smartphone, internet connectivity, and the ability to operate a computer. Exclusion criteria will include breast cancer survivors with active cancer, morbid obesity (BMI > 40 kg/m^2^), those on weight-loss medication, pregnant individuals, or those with any medical condition that would preclude participation in a diet and exercise program.

The duration of the NutriLife intervention will be 24 weeks, combining individual, group, and digital sessions (with the use of asynchronous methods) grounded in the principles of Bandura’s Social Cognitive Theory (SCT) [22]. Therefore, the content of the weight loss intervention will be based on the identified theoretical constructs and the behavioral change techniques (BCTs) that were identified by the systematic review. The sessions will mainly focus on the determinants that have been recorded through the two qualitative studies (focus groups with breast cancer survivors and interviews with health professionals). Those determinants are categorized into three main factors—individual, social, and environmental—which inform the intervention’s contents, enhanced by BCTs such as social support, education, personalized diet plan, advice and goals, flexible recommendations, and a follow-up plan.

Both individual and group sessions will be led by registered dietitians/nutritionists (RDNs). Group sessions will involve both RDNs and clinical psychologists. A team consisting of RDNs, oncologists, clinical psychologists, and physical activity (PA) coaches will facilitate the digital sessions.

Over the 6-month intervention period, the participants will receive 5 individual counseling sessions, 4 group sessions, and 15 digital sessions. The contents of the intervention are available in Supplemental Material S1.

#### 2.1.2. Intervention Targets and Outcomes

The primary outcome of the 6-month intervention is to achieve and maintain a 10% reduction in initial body weight. To achieve the targeted weight loss, the participants will receive a personalized diet plan aimed at reducing energy intake, creating an energy deficit of 500 to 1000 kcal per day, based on their initial body weight and energy requirements. The diet plan will be formulated according to Mediterranean diet guidelines, emphasizing a plant-based approach that is rich in fruits, vegetables, and fiber, while restricting sugar, alcohol, and processed meats/foods. Individualized guidance will be provided to facilitate the achievement of dietary goals.

In relation to the target of physical activity, it is advised that each exercise session exceed 30 min, preferably conducted daily, with a gradual increase in duration over time. The participants will be encouraged to engage in either organized workout sessions or increase their regular physical activity, such as walking or swimming. The participants will be provided with a pedometer to monitor their daily steps. The number of steps and the minutes spent participating in physical activity will be compared to the weekly target.

The participants will be encouraged to submit their weight loss, dietary, and physical activity records on a weekly basis to self-monitor their weight status and lifestyle modification progress.

Furthermore, individuals will receive written materials in line with the World Cancer Research Fund (WCRF) recommendations regarding nutrition and physical activity for breast cancer survivors [1].

#### 2.1.3. Plan for Quantitative Assessment of NutriLife Intervention Effectiveness

The impact and outcome evaluation of the NutriLife intervention will be tested through a randomized controlled trial (RCT), using an intervention and a control group.

**Figure 1:**
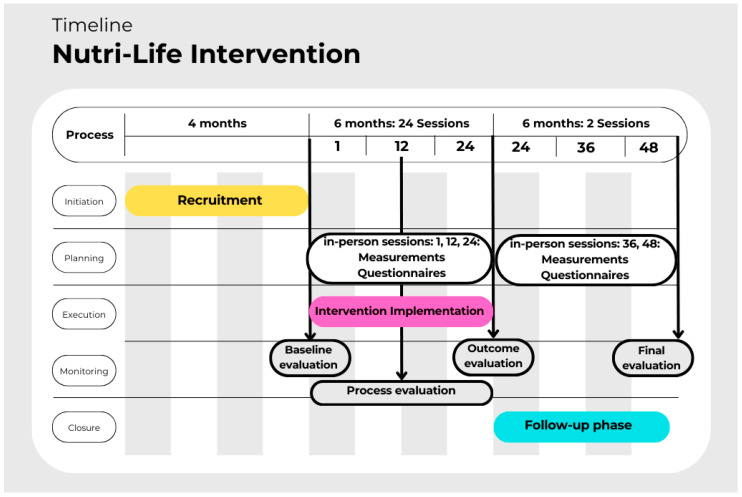
Timeline of the NutriLife intervention.

The NutriLife intervention will be implemented and evaluated according to the timeline presented in Figure 1.

#### 2.1.4. Sample Size and Randomization

The sample size calculation foreseen for the RCT is presented in Supplemental Material S2. Briefly, after assuming that in the intervention-guided arm of our study, 25% of the participants will lose at least 10% of their baseline body weight, while in the control group, 4% of the participants will lose at least 10% of their baseline body weight, as well as after assuming a 10% drop-out rate during the 6-month period, a sample size of 92 participants (46 participants in each arm) is deemed sufficient to detect a statistically significant difference at a 95% confidence level with 80% power [23].

Women will be randomly assigned to one of two study groups using a random permuted block design. To ensure the homogeneity of the groups, the research group will consider multiple factors such as age, menstruation, education level, financial status, employment status, marital status, and place of residence. Research staff responsible for assessing forms and inputting data will be unaware of the participants’ assigned study group, thereby ensuring blinding. Subsequent to randomization, a member of the research team will contact each participant to inform them as to which group they were assigned and to arrange the first meeting.

#### 2.1.5. Measurements and Procedures

Measurements will be conducted in laboratories and clinics during the intervention phase at weeks 1, 12, and 24 and at the end of the follow-up phase at week 48. During the baseline visit (week 1), measures of body weight, height, waist, and hip, in conjunction with a body composition analysis and all questionnaires, will be collected. In the middle of the intervention at week 12, only body weight and a satisfaction survey will be assessed. Body weight, waist and hip circumference, body composition analysis, and all baseline questionnaires will be evaluated at the final visit (week 24).

Initially, a questionnaire relating to the medical history of the participants will be administered, following another survey focusing on sociodemographic characteristics.

Considering dietary intake, a 24 h dietary recall and a 120-item food frequency questionnaire (FFQ) will be used. Adherence to the Mediterranean diet will be assessed using MedDietScore [24].

The assessment of physical activity levels will be conducted using the modified International Physical Activity Questionnaire (IPAQ).

The SF-12 questionnaire will be used for the evaluation of mental and physical health, as both factors significantly influence an individual’s overall quality of life. Additionally, the Impact of Weight on Quality of Life–Lite (IWQoL-Lite) questionnaire will be utilized to evaluate the quality of life experienced by breast cancer survivors.

The assessment of sleep will be conducted using the Pittsburgh Sleep Quality Index.

The Body Image and Relationships Scale (BIRS) and the 4-item Concerns about Recurrence Questionnaire (CARQ-4) will be measured.

Psychological outcomes, particularly self-efficacy as measured by the Rosenberg scale, depression as assessed by the CES-D (Patient Health Questionnaire-8), and anxiety as evaluated by the 20-item state-trait anxiety index, will also be included in the assessment.

In the context of the study, the attendance rates of individuals participating in in-person and group sessions, the frequency of weekly weight, diet, and exercise log submissions, the level of adherence to diet and activity goals on a weekly basis, and the extent to which the participants comply with follow-up sessions will be examined. Moreover, the participants’ (breast cancer survivors’) level of adherence to the intervention, perceived barriers and facilitators for its implementation and satisfaction, as well as all intervention costs will be recorded through questionnaires.

#### 2.1.6. Ethical Approval

This study adhered to the Declaration of Helsinki and European General Data Protection Rules (GDPR) and was approved by the Bioethics Committee of the Department of Nutrition and Dietetics at the University of Thessaly (63/29 October 2024).

#### 2.1.7. Study Design Justification

The current manuscript presents the development and acceptability phase of the NutriLife intervention, as outlined in the updated MRC framework for complex interventions. Within this framework, intervention development is recognized as a distinct and necessary phase of research, particularly when addressing complex behaviors and settings. This study integrates evidence-based design, stakeholder co-creation, and theoretical evaluation using the Theoretical Framework of Acceptability (TFA), enabling refinement before implementation. As part of this phased approach, a randomized controlled trial (clinicaltrials.gov ID: NCT06577545) was planned to evaluate the intervention’s feasibility, adherence, and clinical outcomes.

### 2.2. Qualitative Assessment of the NutriLife Intervention Process Based on Stakeholders’ Perceived Acceptability

#### 2.2.1. Participants

The study participants consisted of (i) female breast cancer survivors aged 18–65 years, with a BMI of 25–40 kg/m^2^, and no active cancer therapy or ongoing treatment, except for hormonal or immune therapy, and (ii) dietitians/nutritionists with experience in the care of breast cancer patients/survivors.

#### 2.2.2. Procedure

Breast cancer survivors were recruited after signing an informed consent form through cancer non-profit organizations by means of announcements on their social media platforms in September and October 2024. A voluntary response sample technique was implemented, wherein women interested in participating in the study were invited to contact M.P., who provided information on the objectives and processes. A total of 22 eligible breast cancer survivors participated, and to attain data saturation, four focus groups were formed, each consisting of 5–7 participants. Data saturation was reached when no new themes emerged during the fourth focus group session. The term “data saturation” describes the number of focus groups necessary to achieve a reliable comprehension of theme exhaustion and diversity within the data collection. It originates from grounded theory but is frequently applied in many qualitative research designs [25].

The collaboration of dietitians/nutritionists in this study was requested through an invitation letter sent to hospitals and local networks that specialize in breast cancer care. Five dietitians/nutritionists accepted the invitation, signed an informed consent form, and were contacted to acquire the necessary information and to arrange an online interview.

The research team generated an interview guide for each group of participants (Supplementary Material S3). The semi-structured interviews were grounded in the Theoretical Framework of Acceptability (TFA), and the findings were analyzed for the seven components of the framework [18]. The TFA defines acceptability as “a multi-faceted construct that reflects the extent to which people delivering or receiving a healthcare intervention consider it to be appropriate, based on anticipated or experienced cognitive and emotional responses to the intervention” and can be assessed before utilization of the intervention (prospectively) or after utilization (retrospectively) [18]. We followed the seven component constructs of the TFA to design the semi-structured interviews’ schedule: affective attitude, burden, perceived effectiveness, intervention coherence, opportunity costs, self-efficacy, and ethicality (Table 1).

All participants were informed of the study’s objectives and purpose, signed the informed consent form, and provided permission for video recording. The assigned consent form was electronically sent to the researcher (M.P.) before their involvement in the study. Participation in the study was entirely voluntary, and the participants were permitted to withdraw at any time. To protect the personal information of each participant, data were anonymized and each participant received a unique code. Every breast cancer survivor participating in the focus groups was offered a complimentary 60-min consultation with the researcher (M.P., a registered dietitian/nutritionist with a master’s in nutrition) and an individualized nutrition plan.

#### 2.2.3. Data Collection

Focus groups and interviews were conducted using an online video conferencing platform (ZOOM). This method was preferred because the participants resided in various locations across Greece, making an in-person meeting infeasible. Prior to the focus groups and interviews, the moderator (M.P.) was comprehensively informed about the intervention and its components through a PowerPoint presentation, ensuring that each session lasted between 60 to 90 min, depending on the number of participants, while each interview with the dietitians/nutritionists lasted approximately 30 min. Both focus groups and interviews were semi-structured and guided by a predetermined list of topics in alignment with the TFA guide (Supplemental Material S3). Every meeting was videotaped and transcribed verbatim by M.P. All transcripts and video recordings were stored in a password-protected university computer in the Lab of Nutrition and Clinical Dietetics at the University of Thessaly.

Basic sociodemographic and medical history data, including age, weight, and height; educational status; region of residence; marital status; number of children; years of survivorship; type of breast cancer; medication; and exercise level, were collected by breast cancer survivors, whereas only the gender, years of experience, health service, and qualifications of dietitians/nutritionists were gathered.

#### 2.2.4. Data Analysis

All transcripts were examined in conjunction with the original video recordings to ensure their accuracy. Data familiarization was accomplished through several readings of the transcripts, followed by a deductive thematic analysis, as described by Braun and Clarke [26]. The transcripts were read by two researchers, and a preliminary coding framework was developed after the line-by-line coding of the transcripts. Preliminary coding was based on the semi-structured interview questions explicitly aligned with the seven TFA constructs. The initial codes and collected data were further analyzed for potential themes within the TFA constructs.

#### 2.2.5. Ethical Approval

This study adhered to the Declaration of Helsinki and European General Data Protection Rules (GDPR), approved by the Bioethics Committee of the Department of Nutrition and Dietetics at the University of Thessaly (63/29 October 2024). All participants in the acceptability study were informed about the purpose and objectives of the study and signed an informed consent form prior to their enrollment.

## 3. Results of the Qualitative Study

The anthropometric, demographic, and disease characteristics of the breast cancer survivors are shown in Table 2. The majority of the participants were married (81.8%), with more than 14 years of education (77.3%), and employed (68.2%). According to their medical history, most of them identified their tumor at stage II (31.8%) or stage III (40.9%); they were positive for hormonal receptors (86.4%) and were currently receiving hormone replacement therapy (72.7%). The 22 participants had a mean age of 48.1 years, a current mean BMI of 30.1 kg/m^2^, and a mean BMI of 27.8 kg/m^2^ at the time of diagnosis. They engaged in regular exercise at a rate of 45.4%, while 36.4% were inactive.

The five dietitians/nutritionists, comprising three males and two females, had a mean age of 44.5 years. All participants held a postgraduate degree and possessed over 10 years of experience. Three worked in public hospitals, with one also engaged in freelance work, while one was solely a freelancer, and another was involved in research/clinical practice (Supplemental Material S3).

The focus groups and interviews were analyzed separately; nonetheless, the results are presented collectively in this section. Themes derived from focus groups and interviews are presented in Table 3, along with quotes and descriptions demonstrating how qualitative data informed the intervention. In brief, based on the seven component constructs of TFA, the results of this study indicated the following:

1. Affective Attitude: This program was perceived as familiar, easily adaptable, supportive, and feasible. It induced neither stress nor uncertainty. It provided two main advantages: tailoring and the ability to access digital sessions at any time. The sole limitation may have been the duration of program engagement, as a few stakeholders expressed ambivalence regarding the intervention’s duration.
2. Burden: The majority of participants reported that they did not encounter any difficulties in completing the intervention. Only a few participants identified barriers that could impede their efforts, including time constraints, insufficient self-care, inadequate digital literacy, and lack of commitment. Some participants struggled to prioritize their personal needs above those of their families, resulting in a deprivation of essential time for self-care. Another participant expressed doubts about her commitment to the program. Only one dietitian/nutritionist raised concern about the elderly BCS’s capacity to cope with digital literacy.
3. Perceived Effectiveness: The majority of the participants believed that the program could achieve its objectives due to a sufficient time frame for achieving the goals. Furthermore, progressively increased goals may prove helpful and less stressful, while educational materials facilitate concentration on these goals. Group sessions may serve as the primary benefit for robust compliance, while the multidisciplinary team effectively aids in achieving the intervention’s objectives.
4. Intervention Coherence: Both types of participants comprehended the functioning of the NutriLife intervention, as they indicated it is an extensive program that considers every aspect of living, including nutrition, physical activity, and mental health. The cohesiveness and rotation of the sessions were well-structured and beneficial and enhanced security.
5. Opportunity Costs: The participants identified numerous benefits associated with this intervention, including time savings, the establishment of daily routines, enhancement of overall health and wellness, reduction of pain, and improved sleep quality. This program provides specific support and recommendations from experts, thus offering unique information. Ultimately, this program provides dietitians/nutritionists with the knowledge and resources necessary to effectively engage with this distinctive population, while also affording breast cancer survivors the opportunity to return to a state of normalcy.
6. Self-efficacy: Numerous breast cancer survivors expressed uncertainty over their self-efficacy, particularly in relation to their ability to adhere to the study objectives or to manage technological barriers. The majority indicated that support from experts could improve their self-efficacy. One participant expressed her capability to accomplish anything following her experiences, while another remarked that the study framework, together with its motivations and objectives, could facilitate her success. One dietitian/nutritionist stated that his self-efficacy was restricted by his insufficient knowledge, while another asserted that with appropriate training, he was capable of accomplishing it.
7. Ethicality: The majority of breast cancer survivors indicated that this intervention was appropriate and representative for this specific population. The NutriLife intervention may improve and promote health; therefore, it was suggested to be mandatory for all women with overweight or obesity who have undergone treatment for breast cancer. A dietitian/nutritionist asserted that privacy must be prioritized, recommending that group sessions be more suitably conducted in clinics or non-profit oncology organizations to safeguard confidentiality and enhance efficacy.

## 4. Discussion

The present paper outlines the development of the NutriLife intervention and its planned future evaluation, while also examining its perceived acceptability as assessed through a qualitative study. The NutriLife study is a lifestyle intervention with dietetic counseling and digital tools designed by a multidisciplinary group of researchers (dietitians/nutritionists, oncologists, physical activity experts, and clinical and health psychologists) for treating overweight/obesity in breast cancer survivors. Its development followed an evidence-based approach and was guided by the MRC framework and a co-creation procedure with stakeholders (breast cancer survivors and dietitians/nutritionists). Dietitians/nutritionists specializing in cancer care and breast cancer survivors, who participated in interviews and focus groups, respectively, identified some barriers and facilitators regarding the NutriLife intervention. Overall, most stakeholders expressed that the intervention could be effectively implemented in real-world settings, as they found it to be supportive, familiar, and easily adaptable. However, certain barriers must be addressed, including time constraints, insufficient self-care, a lack of commitment, and inadequate digital literacy.

The majority of stakeholders perceived the intervention to be supportive, familiar, and emotionally safe. Several breast cancer survivors reported feeling “not alone” as a result of the group sessions, while the dietitians/nutritionists noticed that the variation in the sessions could enhance the survivors’ adherence due to the close interaction. The therapeutic value of peer support and personalized care is an overlooked but powerful facilitator. A recent systematic review of qualitative research on the barriers and facilitators to weight management interventions in breast cancer patients highlighted the pivotal role of peer support in promoting adherence to such programs [27]. Beyond this, evidence suggests that support from peers, broader social networks, and healthcare professionals—coupled with comprehensive supportive care—positively influences all major determinants of behavior change in breast cancer survivors [28]. Consequently, the incorporation of structured support mechanisms is considered a fundamental component in the design of effective behavior change interventions. Additionally, acknowledging that “one size does not fit all,” the necessity of customizing weight management and physical activity interventions is a critical element in their efficacy [29].

Although a number of breast cancer survivors reported a sense of confidence, others expressed reservations regarding long-term adherence, effective time management, and the use of technology. These findings underscore the importance of integrating confidence-enhancing strategies into the NutriLife intervention, including peer mentoring, individualized coaching, and structured technological orientation. A key element of the NutriLife intervention is its variety of sessions (individual, group, digital), which guarantees numerous advantages. Group sessions facilitate peer mentoring, individual sessions provide personalized coaching, and digital sessions may include technological instruction. A systematic review that compared the effectiveness of individual or group interventions in long-term weight loss found that the mean weight change at the final follow-up was −1.33 kg (95% confidence interval CI: −2.04, −0.62; 10 trials, 2169 participants), favoring group interventions over individual interventions (*p* < 0.001) [30]. These findings are consistent with another review that examined the evidence regarding the efficacy of group versus individual multicomponent lifestyle interventions for weight management [31]. Peer social support significantly facilitates successful weight loss and long-term weight maintenance. The peer support format enables members to offer positive reinforcement for success, thus enhancing their self-efficacy [32]. Conversely, individualized interventions provide tailored guidance that aligns with patient attributes and therapeutic needs [33]. In addition, a recent randomized trial indicated that a remotely delivered asynchronous lifestyle intervention resulted in slightly less weight loss compared to an in-person version; however, it may be more cost-effective and convenient [34]. To address the diverse needs and expectations of this distinct study population, the NutriLife intervention included all types of sessions in its protocol.

To include participants from remote areas of Greece lacking healthcare services (such as islands and isolated regions), the NutriLife intervention is designed to be primarily delivered remotely, utilizing an online video conferencing platform along with user-friendly asynchronous tools like pre-recorded videos, newsletters, and podcasts. These resources do not require specialized software or advanced digital literacy, making them more accessible to the entire population (including BCSs and HPs). Clearly, the intervention cannot reach women with limited online access unless community services provide them with the necessary technology. Therefore, collaboration with community organizations to facilitate technology access is essential to ensure that all individuals can benefit from the NutriLife intervention.

The participants reported that they were either too preoccupied or experiencing difficulty prioritizing their own needs. This statement highlights the necessity for prospective adaptations in the intervention content, such as shorter sessions, asynchronous components, and simplified self-monitoring tools. The participants’ ambivalence was evident in the outcomes of the qualitative study, and this must be considered. The NutriLife intervention’s session content, digital tools, and materials have already been developed; however, based on the aforementioned findings, they must be adjusted to each participant’s needs and preferences.

Prior to implementation, the feasibility and perceived acceptability of the NutriLife intervention were assessed through a qualitative study involving breast cancer survivors and oncology-specialized dietitians/nutritionists, using a co-creation approach aligned with best practices in complex intervention development. Positive evaluations from both groups underscore the value of participatory design and theoretically grounded frameworks—specifically, the updated Medical Research Council (MRC) framework and the Theoretical Framework of Acceptability (TFA). While such frameworks are increasingly applied in lifestyle intervention research [35,36], this study extends their use to a culturally specific Mediterranean healthcare context by combining them with Social Cognitive Theory (SCT) and hybrid delivery methods tailored to the unique needs of breast cancer survivors. Stakeholders highlighted the intervention’s flexibility, accessibility, and user-friendliness, noting that features such as progressive goal-setting, structured educational materials, and asynchronous digital components (e.g., videos, online sessions) promoted engagement while reducing psychological and logistical barriers. These elements, together with minimal technological requirements and low operational costs, were seen as essential for reducing participation barriers—particularly for individuals facing geographic, economic, or time-related constraints. Such design choices enhance user experience and support broader implementation potential. This aligns with recent frameworks on implementability, which emphasize adaptability, cost efficiency, and minimization of end-user burden as critical for real-world sustainability [37]. Furthermore, emerging research on digital health literacy highlights the need to tailor digital interventions to users’ capabilities and perceptions—especially in underserved populations [38]. Accordingly, the NutriLife intervention may serve as a transferable model for lifestyle management in breast cancer survivorship beyond the Mediterranean region.

Investing in the development, feasibility, and evaluation phases of complex interventions is cost-prohibitive and time-intensive; nevertheless, it is an opportunity for improvement that must not be overlooked prior to implementing them in real-world settings. Developers have a better chance of creating an intervention that is well-adopted, appropriate for its context, potentially cost-effective, and prepared for piloting if elements such as needs assessment and problem identification are included in the intervention design [39]. In this perspective, alternative frameworks for developing interventions incorporate data from both literature reviews and clinical experience and provide step-by-step guidance for the co-creation of the content and delivery processes of an intervention before its implementation [40,41]. Thus, engaging stakeholders in intervention development provides several advantages, including enhancing comprehension of time and resource constraints in real-world conditions and facilitating interactions with, understanding, and influencing organizational, professional, and social systems that may be resistant to change [16].

## 5. Strengths and Limitations

The NutriLife intervention was developed through a multidisciplinary, theory-driven, and co-creative approach that aligns with current best practices in complex intervention design. Its integration of the Medical Research Council (MRC) framework and the Theoretical Framework of Acceptability (TFA), in combination with Social Cognitive Theory (SCT), enhances both conceptual rigor and practical relevance.

Specifically, the NutriLife intervention incorporates

(a) a multidisciplinary team of healthcare professionals (dietitians/nutritionists, oncologists, physical activity experts, and psychologists) for its development;
(b) personalized in-person nutritional counseling aimed at enhancing self-efficacy;
(c) group-mediated cognitive behavioral support and peer interaction to foster self-regulation and problem-solving skills;
(d) individualized dietary guidance tailored to patients’ specific needs; and
(e) evidence-based, asynchronous digital tools that support behavioral weight management and promote maintenance of newly acquired behaviors.

Despite these strengths, several limitations must be acknowledged. Recruitment via online platforms likely introduced self-selection bias, favoring participants already motivated toward lifestyle change. The participants were predominantly well-educated and digitally literate, which may limit generalizability to underserved or digitally excluded populations. The remote nature of qualitative data collection may have limited the observation of nonverbal cues. The use of self-reported anthropometric data introduces recall and social desirability bias. Although standard procedures were applied, researcher bias may have influenced the data interpretation. Lastly, the intervention was developed and tested qualitatively within a specific regional and cultural context in Greece; thus, its applicability to other cultural or healthcare systems should be approached with caution until further testing is conducted.

## 6. Future Implications

The forthcoming randomized controlled trial (clinicaltrials.gov ID: NCT06577545) will address several of the current study’s limitations by incorporating broader and more inclusive recruitment strategies and by evaluating the intervention’s feasibility, adherence, and clinical effectiveness. Subgroup analyses will examine differential engagement and outcomes based on socioeconomic status, education, and digital access. While the digital components were intentionally designed to be simple and asynchronous, future refinements may include support for individuals with low digital literacy or connectivity barriers.

Furthermore, emerging research on digital health literacy, such as the work by Chen et al., highlights the need for tailored digital interventions that align with users’ capabilities, preferences, and perceived risks [38]. These insights will inform future iterations of the NutriLife intervention, with the goal of enhancing inclusivity, equity, and sustainability in lifestyle intervention delivery for breast cancer survivors. In the long term, the model established by NutriLife may serve as a transferable framework for similar interventions targeting chronic disease management and survivorship care in other populations.

## 7. Conclusions

This study describes the development and qualitative assessment of the NutriLife intervention, a culturally tailored lifestyle intervention for breast cancer survivors with overweight or obesity. Conducted in the Greek healthcare context and informed by co-creation and established frameworks, the intervention was perceived as acceptable, relevant, and adaptable to real-world settings. While limitations exist, the upcoming randomized trial will further examine its effectiveness, feasibility, and implementation potential. The NutriLife intervention may serve as a scalable model for survivorship care in similar populations.

## Figures and Tables

**Table 1:** Questions related to seven domains in Sekhon’s Theoretical Framework of Acceptability. Acceptability study of the NutriLife intervention.

	Focus Groups with Breast Cancer Survivors: Topics	Interviews with Dietitians/Nutritionists: Topics
1. Affective attitude	How participants feel about taking part in the intervention	How dietitians/nutritionists feel about delivering the intervention
2. Burden	The perceived effort required to participate in the intervention	The perceived effort required to deliver the intervention
3. Perceived effectiveness	How effective could the intervention be	The extent to which the intervention is perceived as likely to achieve its purpose
4. Intervention coherence	To what extent do participants understand the intervention and how it works	To what extent dietitians/nutritionists understand the intervention and how it works
5. Opportunity costs	The extent to which benefits, profits, or values must be given up to take part in the intervention	The extent to which benefits, profits, or values must be given up to deliver the intervention
6. Self-efficacy	Participant’s confidence that they can perform the behaviors required	Dietitians’/nutritionists’ confidence to deliver the intervention
7. Ethicality	The extent to which the intervention fits with their value system	The extent to which the intervention fits with their value system

**Table 2:** Characteristics of breast cancer survivors participating in the acceptability study of the NutriLife intervention.

Characteristics (N = 22)	Number (%)
**Marital Status**	
Married	18 (81.8)
Divorced	2 (9.1)
Single	2 (9.1)
**Education Level**	
Lesser (secondary school)	1 (4.5)
Basic (high school, technical school)	4 (18.2)
Higher (university, master’s, PhD)	17 (77.3)
**Employment Status**	
Working	15 (68.2)
Unemployed	3 (13.6)
Retired	4 (18.2)
**Stage of breast cancer**	
Stage I	3 (13.65)
Stage II	7 (31.8)
Stage III	9 (40.9)
Unknown	3 (13.65)
**Type of treatment**	
Surgery + radiation	3 (13.6)
Surgery + chemotherapy	6 (27.3)
Surgery + chemotherapy + radiation	13 (59.1)
**Hormone receptors**	
Positive	19 (86.4)
Negative	3 (13.6)
Hormone therapy	16 (72.7)
**Exercise**	
Regular exercise	10 (45.4)
Occasional exercise	4 (18.2)
Sedentary	8 (36.4)
Years of survival; mean ± standard deviation	4.3 ± 2.6
Age (years); mean ± standard deviation	48.1 ± 6.2
Weight at diagnosis (kg); mean ± standard deviation	73.5 ± 13.5
Current weight (kg); mean ± standard deviation	79.6 ± 8.8
Height (cm); mean ± standard deviation	162.5 ± 5.9
BMI at diagnosis (kg/m^2^); mean ± standard deviation	27.8 ± 5.1
Current BMI (kg/m^2^); mean ± standard deviation	30.1 ± 3.3

**Table 3:** Themes and quotes derived from interviews with dietitians/nutritionists and focus groups with breast cancer survivors.

ID Number	Excerpt
**1. Affective attitude**	
**Flexibility**	
BCS07	“Being able to watch digital content whenever you want is very facilitating.”
BCS08	“Maybe some weeks I won’t have the time to regularly engage since I have three children and my time is limited.”
DN01	“I think the intervention program sounds fantastic, but I’m concerned about the amount of time required to participate—perhaps it’s too long for them.”
**No stress, no doubts**	
BCS01	“For the beginning, it sounds nice; it’s not a short timeframe, which could be stressful, but it’s enough, and it gives you the time to work with yourself.”
BCS09	“The given time of six months is just perfect—not too long to get stressed out or too short for wondering if I can do it. For me, this timeplan works great.”
**Familiar, easy, supportive, feasible**	
BCS05	“Program seems very familiar to me. Do you know why? After cancer, exercise it’s been in my daily routine. I just eat a lot. Even though I eat healthy, I eat a lot. This is how I deal with stress: I eat, emotionally.”
BCS01	“In general it’s an easy program. If you ask me to join tomorrow, I would say: Let’s go.”
BCS04	“I liked the multidisciplinary approach and the psychologist’s presence; perhaps that’s why our previous attempts didn’t work out. Additionally, it was created exclusively for breast cancer survivors, it’s suitable for us.”
BCS16	“Being with a group, makes me feel a relief. We share the same concerns, so I don’t feel alone anymore.
BCS22	“In group sessions, we are a community and we fight together; it’s not only my fight with my weight; I feel stronger inside the group. We have same issues.”
DN04	“This program is great; it’s easy, realistic and feasible, just the way it should be.”
**Educational materials**	
BCS04	“Educational materials are like stimulant injections until we meet again. They keep us in touch, which is quite helpful.”
DN02	“All of these materials are outstanding! It’s beyond my highest expectations! Excellent work. If you ask me, I’d have to pay to get it.”
**Tailor-made**	
BSC09	“I really like it since the program is designed specifically for breast cancer survivors. We have many similarities and distinctions. So, while the program is targeted at BSC, it is also tailored to my specific needs. This is a significant advantage.”
**2. Burden**	
**No difficulties**	
BCS01	“I don’t believe there is anything too difficult for me in the process.”
BCS07	“I see nothing to be difficult here. Only the begging will be difficult but I’ll get used to it.”
**Time as an inhibitor**	
BCS03	“Time would be the hardest thing for me. Some days I have no time at all. Lack of time worries me and might force me to give up. This might also be an issue with cooking.”
BCS12	“I don’t have time to exercise. Between work, my daughter, and daily life, I don’t have time for myself.”
**Loyalty**	
BCS05	“My main worry is my commitment to the program. It’s not the program; it’s my commitment to it that will guide me.”
DN03	“The level of commitment varies depending on whether the content is free to use or requires payment.”
**I am afraid of myself**	
BCS04	“The issue might be with me. To take care of myself, make time for myself, and be successful at it.”
BCS17	“If we want to work out, we will find the time; but, I don’t care about myself, I don’t put myself first, I don’t think about myself; but, if I won’t do it by myself, who would?”
**Technology**	
DN01	Regarding the digital sessions, I’m not sure whether elderly BCS can manage with this digital materials.”
**3. Perceived effectiveness**	
**Time: Ideal timeframe to succeed and maintain weight loss**	
BCS05	“This is the ideal timeline since it allows you plenty of time to lose and keep weight.”
BCS11	“It’s an ideal period of time because losing weight quickly is unhealthy.”
**Focus on the goals**	
BCS01	“Goals could make things easier: Setting a specific goal and knowing that it’s good for my health, it becomes less stressful and will help me succeed with the program.”
BCS06	“Setting goals that get harder over time has always helped me. And this rotation of the sessions—individuals, group, digital—helps me stay focused to my goals. This is the best way to succeed.”
DN01	“I like that goals are gradually increased; this works well most of the time.” My only worry is that the final goal of 10,000 steps sounds really hard.”
DN05	“I enjoyed the concept with the group sessions; I feel it will be beneficial especially with the physical activity goals; the digital material will enable them to stay in touch with their goals. The sessions are very interesting.”
**Contact**	
DN04	“Digital sessions are really smart since this close contact usually helps on a strong compliance, it’s more than enough.”
DN03	“Group sessions are very important because you can discuss the problem; it’s similar to group therapy, and it’s very effective to share your experiences and listen to others. Additionally, the presence of a psychologist is advantageous because mental health services are needed by this population. Every member of the multidisciplinary team contributes to accomplish the success.”
**4. Intervention coherence**	
**Informative program**	
BCS03	“The program, the sessions, the educational materials, and the specialists are all extremely informative, which makes you feel safe. The program’s entire structure is very helpful.
**Complete program**	
BCS05	“It’s a multidisciplinary program, with dietitians, psychologists, oncologists, gym teachers which makes it a complete intervention program.”
BCS18	“It’s a complete plan that addresses all aspects, including mental health, physical activity, and nutrition.”
**Well-organized program**	
DN03	“This program is very well structured and very well organised.”
**5. Opportunity costs**	
**Power of habit**	
BCS07	“I expect all these changes to become my new way of life, to change my perspective on nutrition and exercise.”
BCS18	“There is enough time to make changes, get used to them, and make them a lifestyle.”
**Co-morbidities**	
BCS02	“It will also help me with my diabetes; my diabetologist will be so pleased.”
BCS10	“Apart from losing weight, I will also help my pancreas problem, which is extremely significant to me.”
**Sleeping well, without pain**	
BCS11	“I’ll try to reach the exercise targets because I was sleeping at night and didn’t have joint pain when I was exercising.”
**Credible sources**	
BCS01	“I enjoy the educational material with videos and tips. Who among us hasn’t looked anything up at the internet at some point? Isn’t it great that this information comes from experts?
BCS17	“It’s a great program because it has specific guidance and recommendations from specialists, which is very important.”
DN02	“You give me the right tools and resources to work with this population! It’s incredible.”
**Normalcy**	
BCS08	“When I start exercising again and eating better, normalcy will return.”
**Approaching cancer**	
DN01	“Dietitians sometimes hesitate to work with cancer survivors; if you provide them all this materials and with the proper training from you, I believe you greatly assist them.”
**Time-saving**	
DN03	“It is very important that there are online sessions, and you don’t have to be there for all of them in person. With all the traffic, parking, and kids, it’s easy and saves time.”
**6. Self-efficacy**	
**Cheating**	
BCS03	“The most challenging goal for me is to lose weight since I usually cheat, whether it’s because I want to try something new, it’s a special occasion, or I want dessert. In my social life food matters. Setting goals for my diet is almost impossible for me.”
BCS09	“Changing some of my harmful daily habits, like nibbling, it’s the hardest for me. However, I can be a soldier if I really want to.”
**Support**	
BCS04	“We have been under lot of pressure; food is an outlet and to cope needs help and support. Therefore, it is absolutely essential that you have a psychologist on your team.”
BCS10	“The most important to me is the support I get from the program’s dietitians. I need someone to help me, to guide me, to check me; else I can stray from the target.”
BCS11	“I can do it if I put it in my mind, with the support of the dietitians and psychologists and the group sessions with other breast cancer survivors, I definitely can do it.”
DN01	“Great! With the right training from you, I think it will work fantastic.”
**Context**	
BCS09	“It’s a matter of personality. For me to be successful, I need a framework. Motivated with goals, the program suits me perfectly.”
DN03	“The educational materials are very helpful, but each dietitian works in their own unique way. The materials should be flexible enough to fit their needs. To be able to adopt these tools rather than replicate them.”
**Technology**	
BCS08	“I don’t check my emails very often because I get a lot of them every day, and most of them are useless. I would rather use social media.”
**I can do everything**	
BCS13	“I don’t have an easy life; I work in the fields, so I can try anything and accomplish it.”
**Knowledge**	
DN02	“I am not knowledgeable enough about interventions.”
**7. Ethicality**	
**Representative for BCSs**	
BCS03	“This program is for people who have survived breast cancer. All of the participants have the same problems and take similar medication. It is an intervention that represents all of us.”
**Requirement for BCSs**	
BCS04	“It’s a targeted program, and since cancer is a chronic illness, we should walk with cancer, like people with diabetes. This program ought to be mandatory for all breast cancer survivors.
**Promote**	
BCS02	“I don’t feel bad at all; in fact, I think this program benefits and advances us.”
BCS10	“It’s an honour and a pleasure to be a part of this kind of program for BCS.”
**Privacy**	
DN05	“Because of the privacy, I’m not sure if the group sessions could take place in a private dietitian’s office. Since you want to be profitable and effective, I think it’s best to work in clinics or non-profit anticancer organisations.”

*BCS = Breast Cancer Survivor; DN = Dietitian/Nutritionist.*

## Data Availability

The data presented in this study are available on request from the corresponding author. The data are not publicly available due to ethical restrictions.

## Supplementary Materials

The following supporting information can be downloaded at https://www.mdpi.com/article/10.3390/healthcare13141683/s1: Supplementary Materials S1, S2, and S3. Refs. [42,43,44,45,46] are cited in Supplementary Materials.

## Abbreviations

The following abbreviations are used in this manuscript

BCS	Breast Cancer Survivor
MRC	Medical Research Council
TFA	Theoretical Framework of Acceptability
SCT	Social Cognitive Theory
PA	Physical Activity
RDN	Registered Dietitian/Nutritionist
WCRF	World Cancer Research Fund
