# Supplementary material for: Formative Development and Acceptability of a Lifestyle Weight Management Intervention for Breast Cancer Survivors in Greece: The NutriLife Study
Source: Healthcare (Basel). 2025 Jul 12;13(14):1683. doi: 10.3390/healthcare13141683 (PMC12294187; doi:10.3390/healthcare13141683)
Supplement: Supplementary file 1 [file healthcare-13-01683-s001.zip › SM/SM2 (Sample size justification).pdf]

Sample size justification

As per the investigation conducted by Stolley et al. (2017), which explored the impact of the Moving Forward weight loss intervention on African American breast cancer survivors (AABCS), early-stage AABCS were randomly assigned to either a 6-month interventionist-guided (N= 125) or self-guided weight loss program (N= 121). The findings revealed weight loss in both groups, with the interventionist-guided group demonstrating significantly greater mean and percentage weight loss at both 6 and 12 months. Specifically, at 6 months, the interventionist-guided group exhibited a substantial difference, losing 3.5 kg (SE= 0.4) compared to 1.3 kg (SE= 0.4) in the self-guided group ($P < 0.001$), and 3.6% (SE= 5.1) versus 1.4% (SE= 3.6) ($P < 0.001$), respectively. Additionally, 44% (95% CI= 36%–53%) of the interventionist-guided group and 19% (95% CI= 13%–27%) of the self-guided group achieved the 5% weight loss goal. In a related context, the POWER-Remote Trial, involving women with stage 0-III breast cancer randomized to a 12-month intervention (POWER-remote; N= 45) versus a self-directed approach (N= 42), reported notable outcomes. At six months, 51% (95% CI= 37%–65%) of women in the POWER-remote arm achieved a weight loss of ≥5% from their baseline body weight, compared to 12% (95% CI= 5%–25%) in the self-directed arm. Furthermore, significant differences were observed between study arms concerning the proportion of women achieving at least a 10% reduction in baseline body weight (22% in the POWER-remote arm, 95% CI= 13%–36%, versus 0% in the self-directed arm, 95% CI= 0%–8%). It is noteworthy that in the POWER-remote group, the mean weight loss at six months was 4.6 kg (SD= 4.8 kg), contrasting with a mean weight loss of 0.5 kg (SD= 3.3) at six months in the self-directed arm.

Moreover, drawing from the LISA Trial, where postmenopausal women diagnosed with breast cancer were randomly assigned to either the Mail-based intervention group (N= 167) or the Individualized Lifestyle Intervention group (N= 171), a statistically significant distinction emerged between these cohorts. This distinction was evident in both the absolute weight change from baseline to 6 months (Mail-based intervention group: Mean (SD)= -0.6 (4.1) kg, as opposed to the Individualized Lifestyle Intervention group: Mean (SD)= -4.3 (4.1) kg) and the relative weight change from baseline to 6

months (Mail-based intervention group: Mean (SD)= -0.7% (4.9%), compared to the Individualized Lifestyle Intervention group: Mean (SD)= -5.3% (4.9%)). Finally, the Living Well after Breast CancerTM Pilot Trial, a randomized controlled trial featuring two arms—a 6-month telephone-delivered behavioral weight loss intervention group (N= 45) and a usual care group (N= 45)—yielded noteworthy results. The behavioral weight loss intervention group demonstrated a statistically significant mean weight change from baseline to 6 months, registering at –4.7 (-6.1, -3.3) kg, equivalent to -5.7% (95% CI= -7.4%, -4.0%) of their initial weight. In contrast, the usual care group exhibited a smaller and non-significant mean weight change from baseline to 6 months, measuring at –1.1 (--2.7, 0.4) kg, or -1.3% (95% CI= -3.2%, +0.5%).

Based on the above-mentioned studies, and more specifically on the POWER-Remote Trial, after assuming that in the intervention- guided arm of our study 25% of the participants will lose at least 10% of their baseline body weight, while in the control group 4% of the participants will lose at least 10% of their baseline body weight, as well as after assuming a 10% drop- out rate during the 6-months period, **a sample size of 92 participants (46 participants in each arm)** is deemed sufficient to detect a statistically significant difference at a **95% confidence level** with **80% power** (Wang & Chow, 2007).

References

1. Stolley M, Sheean P, Gerber B, Arroyo C, Schiffer L, Banerjee A et al (2017) Efficacy of a weight loss intervention for African American breast cancer survivors. J Clin Oncol. 35(24):2820– 2828. https://doi.org/10.1200/JCO.2016.71.9856.
2. Santa-Maria CA, Coughlin JW, Sharma D, Armanios M, Blach- ford AL, Schreyer C et al (2020) The effects of a remote-based weight loss program on adipocytokines, metabolic markers, and telomere length in breast cancer survivors: the POWER-remote trial. Clin Cancer Res 26:3024–3034. https://doi.org/10.1158/1078-0432.ccr-19-2935.
3. Goodwin PJ, Segal RJ, Vallis M, Ligibel JA, Pond GR, Robidoux A et al (2014) Randomized trial of a telephone-based weight loss intervention in postmenopausal women with breast cancer receiv- ing letrozole: the LISA trial. J Clin Oncol 32(21):2231–2239. https://doi.org/10.1200/jco.2013.53.1517.
4. Reeves M, Winkler E, Mccarthy N, Lawler S, Terranova C, Hayes S et al (2017) The living well after breast cancerTM pilot trial: a weight loss intervention for women following treatment for breast cancer. Asia-Pac J Clin Oncol 13:125–136. https://doi.org/10. 1111/ajco.12629.
5. Wang, H. and Chow, S.-C. (2014). Sample Size Calculation for Comparing Proportions†. In Wiley StatsRef: Statistics Reference Online (eds N. Balakrishnan, T. Colton, B. Everitt, W. Piegorsch, F. Ruggeri and J.L. Teugels). https://doi.org/10.1002/9781118445112.stat07091.
