# Supplementary material for: Formative Development and Acceptability of a Lifestyle Weight Management Intervention for Breast Cancer Survivors in Greece: The NutriLife Study
Source: Healthcare (Basel). 2025 Jul 12;13(14):1683. doi: 10.3390/healthcare13141683 (PMC12294187; doi:10.3390/healthcare13141683)
Supplement: Supplementary file 1 [file healthcare-13-01683-s001.zip › SM/SM3-Interview Guides.pdf]

Table 1: BCS' Interview Guide in alignment with the TFA guide

Interview topics	Questions
Affective attitude	How did you feel about the goals regarding improving eating habits?<br>How did you feel about the goals regarding increasing physical activity?<br>Did you like the intervention material? The videos, the podcasts, the newsletters? What do you like most and what do you like least?<br>Would you prefer any changes to the material?
Burden	What do you think about the duration of the sessions and the entire intervention program?<br>What factors could make it difficult for you to complete the program?
Ethicality	How suitable is this intervention for women with breast cancer?<br>Do you believe there are any ethical consequences to the intervention that we should consider?
Intervention Coherence	How well do you believe you perceive the intervention's contents?<br>Do you have any questions concerning the content or the program's implementation?
Opportunity costs	In what ways do you believe the intervention could help you?<br>Give us examples of how the intervention will benefit you.
Perceived effectiveness	What are your expectations from the program?<br>How could it differ from other methods of losing weight in women with breast cancer?
Self-efficacy	How likely is it for you to complete the program?<br>How easy or difficult is the program for you?
General acceptability	What is your level of acceptance for this intervention?<br>What do you enjoy and dislike the most?<br>What else do you want us to include or exclude from the program?

Table 2: Dietitians' Interview Guide in alignment with the TFA guide

Interview topics	Questions
Affective attitude	What is your opinion of the program's duration?.<br>What were your views on the objectives for enhancing dietary habits?<br>What was your impression of the objectives focused on increasing physical activity?<br>Please provide us with information regarding the intervention program's structure (which consists of rotation of weekly sessions from individual to group and digital sessions?)<br>Pleased you with the intervention material? The podcasts, the videos, and the newsletters?<br>What are your favourite and least favourite things?
Burden	In what ways do you believe the program can facilitate or complicate your work with patients?<br>How would it change your approach to patient care?
Ethicality	How suitable is this program for dietitians?
Intervention Coherence	To what extent is the intervention program completely understandable?<br>Which components of the program are unclear to you?
Opportunity costs	Have you any reason to believe that the intervention will impose an additional burden on you?<br>What advantages does this particular program give you in your patient care?

Perceived effectiveness	Does the intervention have the potential to effectively assist women with breast cancer in reducing their body weight?<br>Compared to other methods of weight loss for women with breast cancer, how could it potentially be more effective?
Self-efficacy	What is your level of confidence regarding the intervention program?<br>How comfortable are you with putting it into practice with your patients?
General acceptability	What are your favourite and least favourite components of the intervention?<br>Are there any modifications you would recommend?<br>If so, would you suggest it to other healthcare professionals?

Table 3: Dietitians' characteristics (n=5)

		N (%)
Age (mean)		44.4 (7.3) [a]
Gender	Male	3 (60)
	Female	2 (40)
Health service	Public	2 (40)
	Public and freelance	1 (20)
	Freelance	1 (20)
	Research/clinical practice	1 (20)
Experience	<10 years	0 (0)
	10-20 years	3 (60)
	>20 years	2 (40)
Qualifications	Professional qualification	0 (0)
	University Degree	0 (0)
	Postgraduate degree	5 (100)

[a]Standard Deviation
