# Supplementary material for: Formative Development and Acceptability of a Lifestyle Weight Management Intervention for Breast Cancer Survivors in Greece: The NutriLife Study
Source: Healthcare (Basel). 2025 Jul 12;13(14):1683. doi: 10.3390/healthcare13141683 (PMC12294187; doi:10.3390/healthcare13141683)
Supplement: Supplementary file 1 [file healthcare-13-01683-s001.zip › SM/SM1-Sessions.pdf]

The NutriLife intervention sessions are shown in the following table.

Table 1: The NutriLife-intervention sessions.

Number	Type of session	Description
Session 1	individual	Measurements, questionnaires, Motivational Interviewing, personalized goal-setting, individualised diet and advices, material for self-monitoring (e.g. diet, exercise and weight logs).
Session 2	digital	4 videos with RDNs, oncologists, psychologists and PA-coaches, about motivation and the importance of adopting healthier lifestyles.
Session 3	digital	Podcasts produced by RDNs focusing on meals and snacks. The quality and the quantity.
Session 4	digital	Newsletters created by RDNs focus on super market visits, the preparation of shopping lists, the organisation of daily meals (emphasising the importance of planning), the adoption of healthy cooking techniques, and the exploration of various recipes.
Session 5	group	RDNs will help with problem-setting and solving, while psychologists will enhance facilitators and eliminate barriers to change.
Session 6	individual	Review of the diet plan, goals and reinforcement by RDNs.
Session 7	digital	Videos by RDNs about time management, commitment, motivation and practical advices.
Session 8	digital	Videos with home-based exercises by a PA-coaches.
Session 9	digital	Podcast by RDNs on social support (family, friends, colleagues). Focus on the environment.
Session 10	digital	Newsletters developed by RDNs related to social eating and/or drinking (restaurants, other houses, events), action planning, relapse prevention, stimulus control.
Session 11	group	RDNs will assist with problem-setting and solving using role playing, while psychologists will strengthen facilitators and eliminate barriers to change.
Session 12	individual	Weighting, satisfaction questionnaire, review progress, problems, and goals. motivation reinforcement, review advice and diet plan, receive/discuss the self-monitoring material.
Session 13	digital	Myths and truths: 3 videos with RDNs, oncologists and psychologists.

Number	Type of session	Description
Session 14	digital	Topics related to evidence-based anticancer foods (alcohol, salt, meat, processed food, sugar, and sweeteners) and how to read food labels, generated by RDNs.
Session 15	digital	Video by RDNs about energy-balance: caloric content of foods and drinks and energy expenditure (physical activity and sedentary behaviour).
Session 16	digital	Video created by RDNs addressing harmful dietary practices and the presentation of unhealthy diets along with their health impacts.
Session 17	group	RDNs will deal with problem-setting and problem-solving, while psychologists will handle with the change process.
Session 18	individual	Fail to plan, plan to fail.<br>Diet plan about weight maintenance and discuss about the maintenance phase.
Session 19	digital	Maintaining the changes: 4 videos with RDNs, oncologists, psychologists, and PA-coaches (providing reinforcement about the lifestyle changes).
Session 20	digital	Topics developed by RDNs focus on body weight maintenance, strategies for achieving this goal, the importance of self-monitoring, behavioural techniques, potential scenarios and their management, as well as practical advice.
Session 21	digital	Video with psychologists on emotional eating: examining the influence of habits and strategies for breaking the cycle; prioritising personal well-being without feelings of guilt; addressing negative thoughts; exploring the body image; and highlighting the necessity of self-commitment.
Session 22	digital	Request from BCS' to upload their videos or queries to specialists with the primary goal of determining their greatest concerns.
Session 23	group	RDNs will focus on the maintenance of healthy body weight, while psychologists will enhance facilitators and eliminate retention barriers.
Session 24	individual	Measurements, questionnaires, motivation reinforcement, receive/discuss the self-monitoring material.

RDNs = Registered Dietitians-Nutritionists; PA-coaches = Physical Activity coaches; BCS = Breast Cancer Survivors;
